# The Effect of Polysaccharides on Preventing Proteins and Cholesterol from Being Adsorbed on the Surface of Orthokeratology Lenses

**DOI:** 10.3390/polym14214542

**Published:** 2022-10-26

**Authors:** Ting-Yao Wu, Lung-Kun Yeh, Chen-Ying Su, Pin-Hsuan Huang, Chi-Chun Lai, Hsu-Wei Fang

**Affiliations:** 1 Department of Chemical Engineering and Biotechnology, National Taipei University of Technology, 1, Sec. 3, Zhongxiao E. Rd., Taipei 10608, Taiwan; 2 Department of Ophthalmology, Chang Gung Memorial Hospital, Linkou, No. 5, Fuxing St., Taoyuan 33305, Taiwan; 3 College of Medicine, Chang Gung University, No. 259, Wenhua 1st Rd., Taoyuan 33305, Taiwan; 4 Institute of Biomedical Engineering and Nanomedicine, National Health Research Institutes, No. 35, Keyan Rd., Zhunan Town 35053, Miaoli County, Taiwan

**Keywords:** alginic acid, lambda-carrageenan, orthokeratology lens, cholesterol adsorption, protein adsorption

## Abstract

The adsorption of tear film compositions such as proteins and lipids on the orthokeratology lenses often lead to infection or corneal damage. In order to investigate whether polysaccharides could prevent tear compositions from being adsorbed on the lens, alginic acid and lambda-carrageenan were added into artificial tear solution. By measuring daily adsorption of cholesterol, lysozyme, and albumin, our results showed that polysaccharides could weakly prevent cholesterol adsorption. In addition, polysaccharides could also reduce albumin deposition over time. Although the effect of polysaccharides on lysozyme adsorption was distinct depending on the concentrations of polysaccharides, the overall results demonstrated that polysaccharides could decrease protein deposition over time. Our results provided an in vitro evidence that polysaccharides may be applied as coating materials on the lens or as the composition of artificial tear solutions or eyedrops, in order to prevent adsorption of tear film compositions that may lead to a reduced incidence of infection or corneal damage for orthokeratology lens wearers.

## 1. Introduction

Myopia is one of the most common refractive errors affecting people [1]. Overnight wear orthokeratology lens (ortho-k lens) is made of rigid gas-permeable material by the design of reverse geometry to temporarily reduce low-to-moderate myopia during sleep, resulting in having normal vision during the day [2,3]. Moreover, ortho-k lenses have been used for myopia control in children [4]. Although the safety of wearing ortho-k lenses has been proven, corneal damage may occur when the lens is difficult to be removed from the eye due to tear composition attached on the lens [5]. Artificial tear solution or eyedrop is commonly used before wear and before removing the lens from the eye, in order to improve dryness and reduce corneal damage. However, there are still many cases where the lens is stuck in the eye and cannot be removed in the morning [6].

The interaction between the ortho-k lens and the tear film is complex. Contact lenses are easily attracted to tear film compositions such as proteins, lipids, and enzymes [7]. We have previously demonstrated that proteins were easily adsorbed on the ortho-k lens in the presence of lipids, and protein deposition was saturated from day 14 while the lens was repeatedly immersed in artificial tear solution and cleaned by the commercial cleaning solution [8]. When adsorbed proteins are not removed completely from the lens, microbial keratitis may occur when there is bacterial colonization [9]. If the adsorption of tear proteins can be reduced, the incidence of infection may also be decreased. In addition, corneal damage may also be avoided with a reduction of protein deposition on the ortho-k lens. However, majority of researches have been focused on removing protein deposition during cleaning steps [10,11,12], but not on preventing proteins from being adsorbed during wearing. In artificial joint system, polysaccharides have been shown to prevent albumin in joint fluid from being adsorbed on the surface [13]. Therefore, it is possible that polysaccharides may play a similar role in preventing proteins in the tear film from being deposited on the surface of the ortho-k lens.

Alginic acid (AA) and lambda-carrageenan (CRG) are natural polymers which have been widely applied in pharmaceutical excipients or medical treatments [14,15,16]. The molecular weights of AA have been reported to be ranged from 32,000 to 400,000 g/mol, and CRG is from 340,000 to 870,000 g/mol [17,18]. The polydispersity index of AA and CRG is both 1.5 [19,20]. Although AA and CRG have been shown to be biocompatible and biodegradable due to formation of hydrogels or hydrocolloids by interacting with water molecules [21], they have not yet been applied for reducing protein adsorption on ortho-k lenses. In this study, we investigated the effect of AA and CRG on prevention of tear proteins and cholesterol by adding them into artificial tear solution. Lysozyme and albumin were used here for protein composition because lysozyme is the most abundant tear protein and the concentrations of albumin are increased during ortho-k lens wear [22,23]. The amounts of cholesterol, lysozyme, and albumin were analyzed daily, and compared between different treatments. Contact angle of lenses after treatments was also measured to analyze the hydrophobicity of ortho-k lenses.

## 2. Materials and Methods

### 2.1. Orthokeratology Lenses, Polysaccharide-Containing Artificial Tear Solution, and Cleaning Solution

The ortho-k lenses used in this study were Brighten 22, and the material generic name was Butyloxyfocon A (Brighten Optix Co., Taipei, Taiwan). The preparation of artificial tear solution with salts and lipids has been published previously [24], and 2 mg/mL of lysozyme (Sigma-Aldrich, St. Louis, MO, USA) and 0.2 mg/mL of albumin (Sigma-Aldrich) were added into salt-lipid mixed solution. After salts, lipids, and proteins were mixed, 225 or 450 mg of alginate acid (AA, molecular weight is 120,000–190,000 g/mol, Sigma-Aldrich) and lambda-carrageenan (CRG, molecular weight is 560,500–580,500 g/mol, Sigma-Aldrich) were added into 100 mL of solution to make polysaccharide-containing artificial tear solution. The 100 mL of cleaning solution contained 0.45 g of AA, 0.2 g of ethylenediaminetetraacetic acid (EDTA, Sigma-Aldrich), 0.75 g Poloxamer-407 (Wei Ming Pharmaceutical Mfg. Co., Ltd., Taipei city, Taiwan), 0.015 g CaCl_2_ (Sigma), 0.15 g KCl (Sigma-Aldrich), 0.45 g NaCl (Sigma-Aldrich), 0.4 g Na_2_HPO_4_ (Sigma-Aldrich), double distilled water in a final volume of 100 mL and pH of 7.4.

### 2.2. Cholesterol and Protein Adsorption Analysis

**Figure 1:**
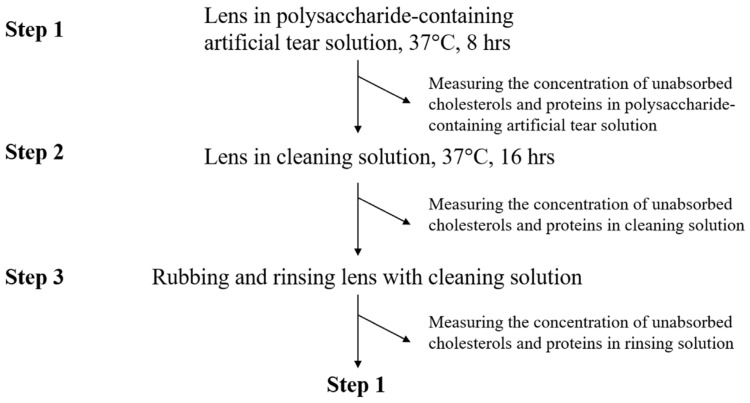
The procedure of cholesterol and protein adsorption analysis. One completed cycle is from step 1 to step 3.

Before analysis, ortho-k lens was first treated by oxygen plasma system (Force State OP300, PFS Co., Ltd., Taipei, Taiwan) at 100 mTorr, 80 W, 10 standard cubic centimeter per minute (Sccm) for 120 s to reduce the surface hydrophobicity. Orthokeratology lens was placed in 2 mL of polysaccharide-containing artificial tear solution, and incubated at 37 °C for 8 h (Step 1 in Figure 1). Polysaccharide-containing artificial tear solution was then replaced by 2 mL of cleaning solution, and the lens was incubated at 37 °C for 16 h (Step 2 in Figure 1). Finally, ortho-k lens was rubbed with fingers in gloves and rinsed by 50 μL of cleaning solution, and placed into a new polysaccharide-containing artificial tear solution (Step 3 in Figure 1). A completed cycle (step 1 to 3) was repeated 3 times (3 days), and cholesterol and protein concentrations in each solution were analyzed.

For cholesterol concentration measurement, the total cholesterol and cholesteryl ester fluorometric assay kit (Elabscience, Houston, TX, USA) was used. The concentrations of cholesterol for standard curve were 0, 2, 5, 10, 15, 20, 25, and 30 μg/mL, and the instructions were followed according to the manufacturer’s procedure. The optical density (OD) value was obtained by an Enzyme-Linked Immuno Sorbent Assay (ELISA) reader with excitation/emission spectra (Ex/Em) at a wavelength of 535 nm/590 nm, and the concentrations of samples could be obtained by the equation of the trendline (Supplementary Materials Figure S1A).The Bio-Rad DC protein assay (Bio-Rad, Hercules, CA, USA) was used for measuring the amount of total protein including lysozyme and albumin in each solution [8], and the OD value was obtained with a wavelength of 280 nm. The concentrations of standard curve were 0, 0.05, 0.1, 0.2, 0.4, 0.8, 1.6, 3.2 mg/mL by diluting protein standard with protein reagent A’ in the Bio-Rad DC protein assay, and the equation could be used for calculating protein concentration of samples (Supplementary Materials Figure S1B). The chicken lysozyme ELISA kit (Cusabio, Houston, TX, USA) and bovine albumin ELISA kit (Cusabio) was used for detecting the concentration of lysozyme and albumin in each solution, respectively. The OD value was then obtained with a wavelength of 450 nm. The concentrations of standard curve for both lysozyme and albumin were 0, 15.625, 62.5, 250, 1000, 4000 ng/mL, and the equation was obtained by Morgan-Mercer-Flodin (MMF) model (Supplementary Materials Figure S1C,D). The deposited cholesterol or protein concentration on the orthokeratology lens after 3 steps calculated as: (the original cholesterol/protein concentration)—(cholesterol/protein concentration in polysaccharide-containing artificial tear solution)—(cholesterol/protein concentration in cleaning solution)—(cholesterol/protein concentration in rinsing solution). Three independent lenses were tested.

### 2.3. Measurement of Contact Lens

After three cycles of the procedure of cholesterol and protein adsorption analysis, water contact angle of each lens was measured using MagicDroplet Contact Angle Meter Model 100SB (Sindatek Instruments Co., Ltd., Taipei, Taiwan).

### 2.4. Statistical Data Analysis

The differences in cholesterol, total protein, lysozyme or albumin adsorption amounts were compared between the lens being immersed in artificial tear solution without polysaccharides (control) and with different concentrations of polysaccharides on the same day. The 2-tailed *t*-test was assessed, and a value of *p* < 0.05 was considered significant.

## 3. Results

### 3.1. Low Concentration of Polysaccharides Could Prevent Cholesterol Adsorption Weakly

**Figure 2:**
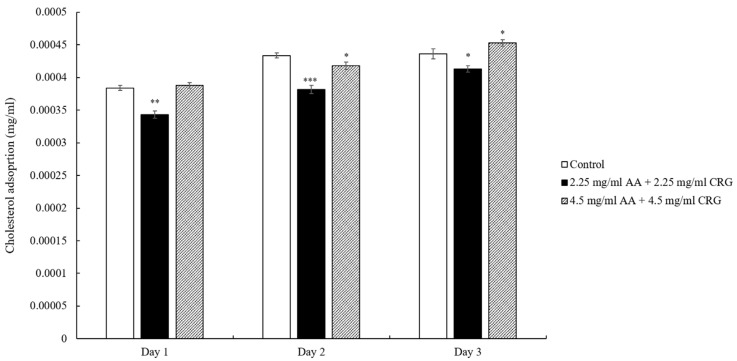
The concentration of cholesterol adsorption on the ortho-k lens when being immersed in artificial tear solution (control, white bars), 2.25 mg/mL (black bars) or 4.5 mg/mL (grey slashed bars) polysaccharides-containing artificial tear solution. * *p* < 0.05, ** *p* < 0.01, and *** *p* < 0.001 when comparing cholesterol adsorption amount in polysaccharides-containing artificial tear solution versus in control on the same day. Error bars represented standard deviation.

When the lens was immersed in artificial tear solution containing 2.25 mg/mL AA and 2.25 mg/mL CRG, the cholesterol deposition was significantly reduced each day comparing with control (Figure 2). When the lens was immersed in tear solution containing 4.5 mg/mL AA and 4.5 mg/mL CRG, the cholesterol adsorption was similar with the control. However, the cholesterol adsorption was increasing daily regardless of the concentration of polysaccharides (Figure 2).

### 3.2. Distinct Effects of Polysaccharides on Lysozyme and Albumin Adsorption

**Figure 3:**
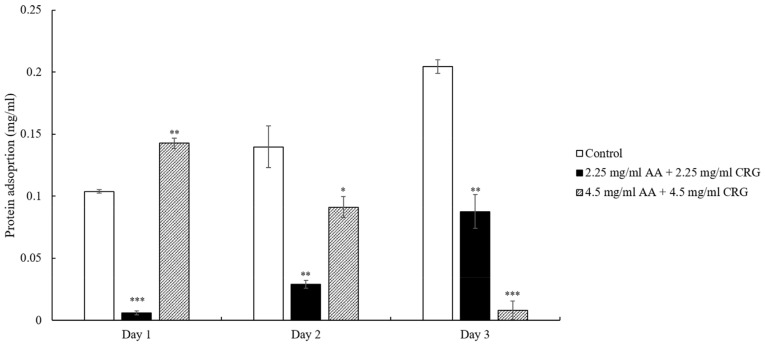
The concentration of total protein adsorption on the ortho-k lens when being immersed in artificial tear solution (control, white bars), 2.25 mg/mL (black bars) or 4.5 mg/mL (grey slashed bars) polysaccharides-containing artificial tear solution. * *p* < 0.05, ** *p* < 0.01, and *** *p* < 0.001 when comparing protein adsorption amount in polysaccharides-containing artificial tear solution versus in control on the same day. Error bars represented standard deviation.

Similar to the cholesterol adsorption result that was displayed in Figure 2, the total protein deposition amounts of lenses immersed in 2.25 mg/mL AA and 2.25 mg/mL CRG artificial tear solution were lower than control although the protein concentration was increased over time (Figure 3). However, the adsorbed protein on the lens in 4.5 mg/mL AA and 4.5 mg/mL CRG-containing tear solution was reduced daily and the protein adsorption amount was the lowest on day 3 (Figure 3). 

**Figure 4:**
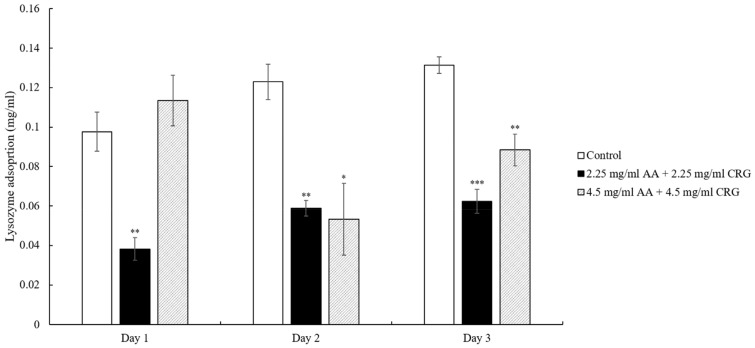
The concentration of lysozyme adsorption on the ortho-k lens when being immersed in artificial tear solution (control, white bars), 2.25 mg/mL (black bars) or 4.5 mg/mL (grey slashed bars) polysaccharides-containing artificial tear solution. * *p* < 0.05, ** *p* < 0.01, and *** *p* < 0.001 when comparing lysozyme adsorption amount in polysaccharides-containing artificial tear solution versus in control on the same day. Error bars represented standard deviation.

The artificial tear solution contained both lysozyme and albumin. To understand the effects of AA and CRG on adsorption of each protein, we then analyzed them separately. When the lenses were immersed in 2.25 mg/mL AA and 2.25 mg/mL CRG artificial tear solution, lysozyme adsorption was significantly lower than control although the amounts were increasing daily (Figure 4). Interestingly, lysozyme deposition amounts on the lens in 4.5 mg/mL AA/CRG-containing artificial tear solution were lower than control on day 2 and 3 but higher on day 1 (Figure 4). During these 3 days, lysozyme deposition did not show specific pattern in the group of 4.5 mg/mL AA/CRG-containing artificial tear solution.

**Figure 5:**
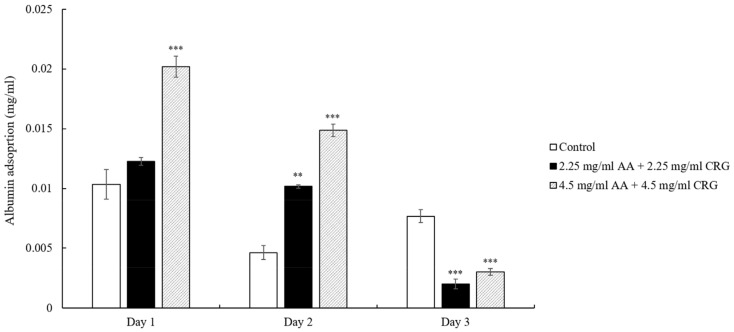
The concentration of albumin adsorption on the ortho-k lens when being immersed in artificial tear solution (control, white bars), 2.25 mg/mL (black bars) or 4.5 mg/mL (grey slashed bars) polysaccharides-containing artificial tear solution. * *p* < 0.05, ** *p* < 0.01, and *** *p* < 0.001 when comparing albumin adsorption amount in polysaccharides-containing artificial tear solution versus in control on the same day. Error bars represented standard deviation.

Different from lysozyme adsorption result, albumin deposition amounts were decreasing on the lens in both 2.25 mg/mL and 4.5 mg/mL AA/CRG-containing tear solutions over time (Figure 5). The albumin adsorption concentrations in polysaccharides-containing tear solution was only lower than it in control on day 3. 

### 3.3. Contact Angle of Lenses Was Reduced after the Treatment of Polysaccharides

After three completed cycles of cholesterol and protein adsorption procedure, water contact angle was measured for each lens. When the lens was not immersed in polysaccharides-containing tear solution, water contact angles were all larger than 74° (Table 1). In contrast, contact angles were smaller than 30° when lenses were treated with 2.25 or 4.5 mg/mL AA/CRG (Table 1).

## 4. Discussion

We analyzed the effects of polysaccharides on the adsorption of cholesterol and proteins on the surface of ortho-k lenses in this study. The result demonstrated that cholesterol was shown to be weakly prevented from being adsorbed on the ortho-k lenses when being immersed in polysaccharides-containing tear solution regardless of the concentrations of AA and CRG (Figure 2). The ortho-k lenses are made mainly with polymethylmethacrylate (PMMA), the surface tends to be hydrophobic although silicone acrylate is added for increasing oxygen permeability and hydrophilicity [25]. It has been shown that the hydrophobic surface of the ortho-k lens may easily attract the hydrophobic site of lipids [26], thus cholesterol adsorption was increasing over time in control. Previous studies have shown that soft contact lenses coated with polysaccharides such as hyaluronic acid or chitosan would increase wetting of the lens, resulting in a decrease of lipid deposition [27]. Therefore, AA and CRG might increase the hydrophilicity of the ortho-k lenses in current study resulting in a weak reduction of cholesterol adsorption. Indeed, the result of water contact angle measurement showed that the hydrophobicity was greatly improved after lenses were immersed in polysaccharides-containing tear solution (Table 1). The effect of lipid deposition on contact lenses is still unclear, a recent research has demonstrated that the amounts of certain lipid deposition may improve comfort during contact lens wear by changing the surface properties of contact lenses [28]. However, whether lipid adsorption on ortho-k lenses may affect the comfort during wear will require further investigation.

The previous study has shown that protein adsorption was greatly increased in the presence of lipids [8], thus reduction of cholesterol in current study indeed affect protein deposition behavior. In general, protein adsorption was reduced in polysaccharides-containing tear solution on day 2 and 3 when comparing with control (Figure 3). However, the protein adsorption pattern was distinct between low and high concentrations of polysaccharides. The total protein adsorption was decreasing over time in 4.5 mg/mL polysaccharides-containing tear solution, possibly due to a great reduction of albumin daily (Figure 5) In contrast, 2.25 mg/mL polysaccharides-containing tear solution mainly prevented lysozyme from being adsorbed dramatically over time (Figure 4). The reason of choosing 2.25 and 4.5 mg/mL of AA and CRG in current study was that these concentrations demonstrated good lubrication and prevention of albumin adsorption in artificial joint system [13]. Higher concentrations of AA and CRG were also investigated in this study. Unfortunately, 9 and 12.5 mg/mL AA and CRG could not be dissolved completely in artificial tear solution. The properties of lysozyme and albumin are very distinct, thus the interaction between different kinds of proteins and polysaccharides was discussed separately.

The isoelectric point of albumin is 5.16 [29], thus albumin carries the same charge with AA and CRG when pH value of the artificial tear solution was 7.4. The interaction between albumin and AA/CRG would not be electric attraction, but biopolymers might be formed [30]. Once biopolymers are formed and adsorbed on the lens, the space on the surface of the lens is occupied resulting in fewer biopolymers on the surface. Therefore, the amounts of albumin deposition were decreasing over time when the surface space was increasingly occupied regardless of the concentrations of polysaccharides. Higher concentrations of polysaccharides might be corresponding to more biopolymers, thus more amounts of albumin adsorption were observed. 

The isoelectric pH of lysozyme is 11.4, and can be easily attracted to the negatively charged surface of the ortho-k lenses [26,31]. Both AA and CRG are also negatively charged that may compete with the surface of ortho-k lens, resulting in preventing lysozyme from being greatly adsorbed on the lens. However, low concentrations of polysaccharides showed a better prevention of lysozyme adsorption than high concentrations. One possibility was that more biopolymers of polysaccharides and albumin were formed when the concentrations of polysaccharides were higher, resulting in fewer polysaccharides in the solution to take lysozyme away from the lens. However, he interaction between lysozyme/albumin and single polysaccharide should be further studied in order to understand how the polysaccharide prevents proteins from being adsorbed on the lens.

**Figure 6:**
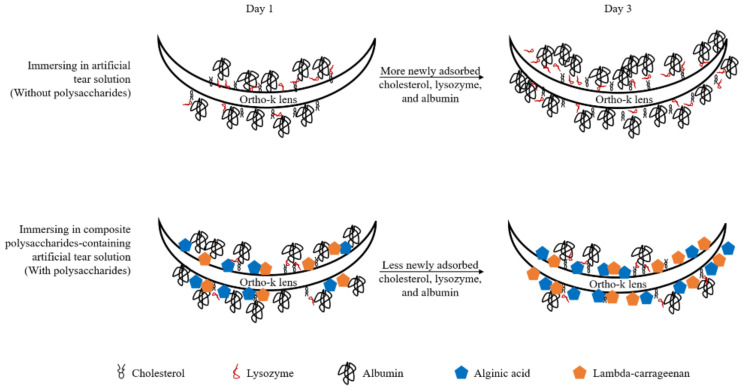
The potential mechanism of how alginic acid and lambda-carrageenan weakly prevent adsorption of cholesterol and strongly inhibit protein (especially albumin) deposition.

In order to reduce protein deposition and cell adhesion on the surface of medical implants, antifouling materials are often used [32]. The antifouling materials can be divided into two classes: polyhydrophilic and polyzwitterionic materials, and polysaccharides are a kind of polyhydrophilic materials [33]. The mechanism of how polysaccharides could be antifouling is to form a hydrated surface, resulting in large repulsion when charged molecules such as proteins approach to it [34]. Therefore, we proposed a potential mechanism of how AA and CRG could prevent cholesterol and protein from being adsorbed on the surface of ortho-k lens (Figure 6). At first day, some AA and CRG were bound to the lens surface to increase hydrophilicity resulting in less cholesterol adsorption than control. However, biopolymers of polysaccharides and albumin might form leading to more albumin adsorption compared to control. At day 3, more polysaccharides were bound to the surface of ortho-k lens to form a hydrated layer. The strong hydration effect of polysaccharides attracts water molecules to the surface resulting in repulsing proteins. Therefore, less cholesterol and proteins could be newly adsorbed.

The limitations of this study were that the methods of detecting cholesterol and protein adsorption were indirect, the concentrations of cholesterol and proteins were calculated from solutions not directly from the lens. Although a recent research has shown to quantitate depositions directly on worn orthokeratology lenses [35], the lens needed to be cut thus the dynamics of depositions would not be observed every day. The methods of measuring the concentration of total proteins, lysozyme or albumin-specific were also different, the sensitivity of different methods might cause some variations. However, the results showed that AA and CRG could weakly prevent cholesterol adsorption. In addition, protein deposition was greatly reduced from the ortho-k lens despite the distinct adsorption patterns of lysozyme and albumin. Our results provided some potential applications, such as coating the surface of ortho-k lenses with AA and CRG or adding AA and CRG into artificial tears or eyedrops for ortho-k lens wearers. By preventing adsorption of cholesterol and tear proteins from the lens, the incidence of infection or cornea damage might be reduced leading to better quality life for ortho-k lens wearers.

## 5. Conclusions

The current study demonstrated that polysaccharides, alginic acid and lambda-carrageenan could weakly prevent cholesterol adsorption on ortho-k lenses when polysaccharides were added in the artificial tear solution. Polysaccharides could reduce albumin deposition over time, while only lower concentrations of polysaccharides could prevent lysozyme from being adsorbed dramatically. Overall, the total protein adsorption was significantly reduced when ortho-k lenses were immersed in polysaccharides-containing artificial tear solution repeatedly. The results provided in vitro evidence for polysaccharides being effective in preventing adsorption of cholesterol and tear proteins on the ortho-k lens that may help to reduce risks for infections and corneal damage.

## Figures and Tables

**Table 1:** Contact angle of each tested lens and averaged contact angle after three cycles of cholesterol and protein adsorption procedure.

Artificial Tear Solution	Contact Angle (°)			
	1	2	3	Mean ± Standard Deviation
Control (without polysaccharides)	76.42	74.86	79.31	76.86 ± 2.26
2.25 mg/mL AA + 2.25 mg/mL CRG	29.32	29.7	24.41	27.81 ± 2.95
4.5 mg/mL AA + 4.5 mg/mL CRG	8.58	21.96	24.51	18.35 ± 8.56

## Data Availability

The data presented in this study are available on request from the corresponding author.

## Supplementary Materials

The following supporting information can be downloaded at: https://www.mdpi.com/article/10.3390/polym14214542/s1, Figure S1: The standard curve for cholesterol (A), total protein (B), lysozyme (C), and albumin (D) used in this study.
