# Supplementary material for: The Effect of Polysaccharides on Preventing Proteins and Cholesterol from Being Adsorbed on the Surface of Orthokeratology Lenses
Source: Polymers (Basel). 2022 Oct 26;14(21):4542. doi: 10.3390/polym14214542 (PMC9658088; doi:10.3390/polym14214542)
Supplement: Supplementary file 1 [file polymers-14-04542-s001.zip › polymers-1999473-supplementary.pdf]

The Effect of Polysaccharides on Preventing Proteins and Cholesterol from Being Adsorbed on the Surface of Orthokeratology Lenses

Ting-Yao Wu [1,+], Lung-Kun Yeh [2,3,+], Chen-Ying Su [1], Pin-Hsuan Huang [1], Chi-Chun Lai [2,3] and Hsu-Wei Fang [1,4,*]

[1] Department of Chemical Engineering and Biotechnology, National Taipei University of Technology. 1, Sec. 3, Zhongxiao E. Rd., Taipei 10608, Taiwan

[2] Department of Ophthalmology, Chang Gung Memorial Hospital, Linkou. No. 5, Fuxing St., Taoyuan 333, Taiwan

[3] College of Medicine, Chang Gung University, No.259, Wenhua 1st Rd., Taoyuan 333, Taiwan

[4] Institute of Biomedical Engineering and Nanomedicine, National Health Research Institutes. No. 35, Keyan Rd., Zhunan Town, Miaoli County 35053, Taiwan.

[+] These authors contributed equally to this work.

* Correspondence: Dr. Hsu-Wei Fang; hwfang@ntut.edu.tw

Figure S1. The standard curve for cholesterol, total protein, lyszoyme, or albumin used in this study. (A) The concentrations of cholesterol standard curve were 0, 2, 5, 10, 15, 20, 25, and 30 μg/ml, and the equation of the trendline was y = 29.854x - 12.688. Y in the equation represents optical density (OD) value, while x represents the concentration of cholesterol. After OD value of the sample was obtained at a wavelength of 535 nm/590 nm (excitaion/emission spectra), the cholesterol concentration of the sample could be calculated according to the equation. (B) The concentrations of total protein standard curve were 0, 0.05, 0.1, 0.2, 0.4, 0.8, 1.6, 3.2 mg/ml, and the OD values were obtained at a wavelength of 280 nm. The equation of the trendline was y = 0.2719x + 0.0043. The standard curve concentrations for lysozyme (C) and albuin (D) were 0, 15.625, 62.5, 250, 1000, 4000 ng/ml, and the OD values were obtained at a wavelength of 450 nm. The equation obtained from lysozyme and albumin standard curve was $y = (20.77 * 1616.39 + 6130.09 * x^{-3.77}) /(1616.39 + x^{-3.77})$ and $y = (0.17 * 5973.87 + 370.85 * x^{-3.45}) /(5973.87 + x^{-3.45})$, respectively. Y in the equation reprensents the concentration of lyszoyme or albumin, while x represents OD value.

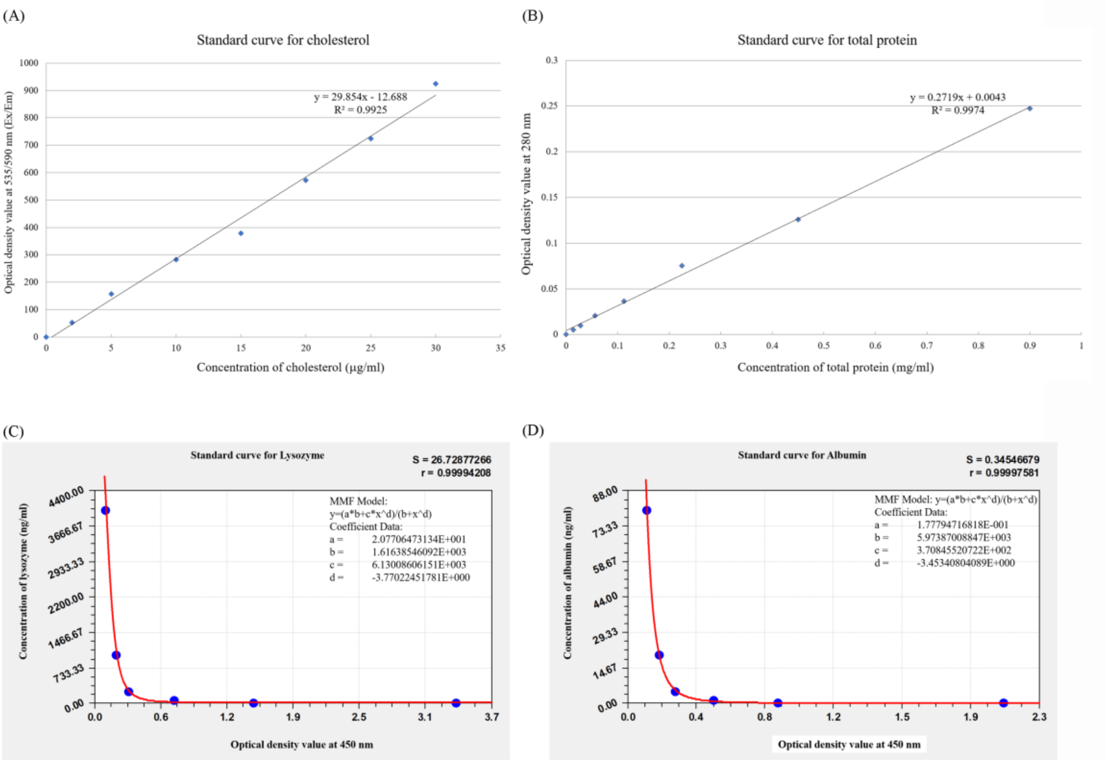
(A)
Standard curve for cholesterol
y = 29.854x - 12.688
R² = 0.9925
Optical density value at 535/590 nm (Ex/Em)
Concentration of cholesterol (µg/ml)
(B)
Standard curve for total protein
y = 0.2719x + 0.0043
R² = 0.9974
Optical density value at 280 nm
Concentration of total protein (mg/ml)
(C)
Standard curve for Lysozyme
S = 26.72877266
r = 0.99994208
MMF Model:
y=(a*b+c*x^d)/(b+x^d)
Coefficient Data:
a = 2.07706473134E+001
b = 1.61638546092E+003
c = 6.13008606151E+003
d = -3.77022451781E+000
Concentration of lysozyme (ng/ml)
Optical density value at 450 nm
(D)
Standard curve for Albumin
S = 0.34546679
r = 0.99997581
MMF Model: y=(a*b+c*x^d)/(b+x^d)
Coefficient Data:
a = 1.77794716818E-001
b = 5.97387008847E+003
c = 3.70845520722E+002
d = -3.45340804089E+000
Concentration of albumin (ng/ml)
Optical density value at 450 nm
